# Another School in the Field

**Published:** 1902-08

**Authors:** 


### ANOTHER SCHOOL IN THE FIELD.

THE advent of a third school in Kansas City, Mo., now makes that municipality the "City of Veterinary Colleges." With two schools already doing work at that centre one is at a loss at this distance to comprehend or conjecture good reasons for this seeming interest in veterinary education. We say veterinary education, not higher veterinary education, for a State that defeats in every session of its Legislature any bill tending to regulate or raise the standard of veterinary education is not destined to sustain any movement of progress along such lines. In that it is a rival two-year school of one already in existence in that city and the outgrowth of internal dissensions in that institution, we can hope for but little good from such undertaking. That it may terminate in the closing of both of these two-year institutions and the strengthening of the one three-year school in that city is to be devoutly desired and prayed for. Not one of these institutions can grow properly in strength, not one can do advanced work, not one can raise the standard of veterinary education as it should be advanced, not one can sustain a proper teaching Faculty or lead to safeguards in regulating the practice in Missouri under the present conditions as they exist, and the rivalry, the competition for support, the jealousies, the intrigue, and ultimate disaster are all to add heavy burdens to veterinary progress and do immeasurable injury to the profession in their own city and State.

